# An Unusual Presentation of Acute Weakness: Acute Inflammatory Demyelinating Polyneuropathy in a Patient with Psychiatric Illness

**DOI:** 10.1155/2018/4065342

**Published:** 2018-09-23

**Authors:** Eric Lombardi, Ryan Misek, Krishna Patel

**Affiliations:** ^1^Franciscan Crown Point Hospital, Crown Point, IN, USA; ^2^Midwestern University, USA; ^3^Chicago College of Osteopathic Medicine, USA

## Abstract

We report an unusual presentation of Guillain-Barré wherein a patient with an extensive history of psychiatric illness had a dream that his legs were crushed in an earthquake and awoke from the dream with paresthesias and rapid paralysis of bilateral lower extremities. This article discusses an atypical presentation of pathology and diagnostic evaluation for a form of Guillain-Barré called Acute Inflammatory Demyelinating Polyneuropathy (AIDP).

## 1. Introduction

Acute Inflammatory Demyelinating Polyneuropathy (AIDP) is the most common variant of Guillain-Barré syndrome in the United States. However, the diagnosis is often difficult and the presenting complaints are easy to dismiss as they are often mistaken for psychosomatic in nature. This case reports a patient with extensive psychiatric illness who presented to the emergency department stating that he had a dream wherein his legs were crushed in an earthquake and he awoke from the dream with paresthesias and rapid paralysis of bilateral lower extremities. This report discusses an atypical presentation of AIDP, the diagnostic process of identifying Guillain-Barré syndrome, a brief literature synopsis of the correlation between psychosis and psychosomatic complaints in patients with Guillain-Barré syndrome, and the importance of evaluating all presenting complaints thoroughly and remembering psychosomatic disorders are a diagnosis of exclusion.

## 2. Case

A 58-year-old male presented to the emergency department via private vehicle with multiple complaints. Complaints included “chest discomfort”, low back pain, shortness of breath, generalized weakness throughout all extremities, and “numbness” of bilateral lower extremities. These symptoms began acutely at around 10 hours prior to arrival when he awoke from a dream. The patient stated during the dream that he was in an earthquake and his legs were trapped and crushed in the earthquake; when he awoke both of his lower extremities were numb and weak. He states that all of his symptoms are progressively getting worse and now he “can't move my legs.”

The patient's past medical history is significant for chronic back pain, anxiety, bipolar disorder, schizophrenia, major depressive disorder, and an episode of previous “paralysis.” The patient states in 1997 that he had a lumbar fusion and while in rehab he “became paralyzed and couldn't move my legs or walk” and that episode of weakness gradually improved and paralysis resolved without any medical intervention. The patient takes a total of 23 for his medical conditions that include zolpidem, methocarbamol, hydrocodone, carisoprodol, alprazolam, and gabapentin.

Vital signs at time of presentation are benign and reveal a temperature of 99.20 F, HR 68, BP 156/84, and Sating 95% on RA. General exam reveals a nontoxic patient in no acute distress, with a disheveled appearance. Neurological exam reveals that patient is alert and orientated X 3, with a GCS of 15, CN 2-12 intact, and 5+ bilateral upper extremity strength, normal finger to nose movement. Decreased bilateral patellar DTRs and decreased bilateral lower extremity strength 4/5. Otherwise physical exam was within normal limits.

A CBC, CMP, CK, sed rate, UA, UDS, Troponin-I, D-dimer, EKG, noncontrast CT's of head and C-spine, and CT of chest/abdomen/pelvis with IV contrast to evaluate aorta with spinal reconstruction were ordered. Pertinent labs results include normal Troponin and D-dimer, calcium 10, sed rate of 9, CK of 437, UDS positive for benzodiazepines and opiates (both of which are chronic medications prescribed to patient), and otherwise unremarkable labs and all imaging within normal limits.

The patient was now reevaluated. We discussed how his evaluation is unremarkable and cannot explain his symptoms. He responds with “Doc, it's getting worse and now I can't move my legs at all.” A repeat neurological exam revealed decreased sensation and bilateral lower extremity strength now 0/5. Pinprick sensation not detected and elicited no movement, despite pinprick drawing blood.

At this point, the evaluation had ruled out electrolyte abnormalities, acute myocardial infarction, pulmonary embolism, aortic dissection, acute CVA, spinal pathology, or tick paralysis as potential causes for the patient's acute weakness. The differential diagnosis continued to include Multiple Sclerosis, Myasthenia Gravis, medication reaction, conversion disorder, and Guillain-Barré syndrome. In light of the patient's psychiatric history, atypical complaints, disheveled appearance, medication list, and history of onset related to a dream in which his lower extremities were crushed, the overall clinical picture suggests a psychosomatic conversion disorder. However, this is a diagnosis of exclusion after CNS, peripheral nervous syndrome, infections, vascular, cardiopulmonary, and other potentially life threatening etiologies are excluded and therefore, the medical decision was made to proceed with lumbar puncture and CSF analysis, as well as MRI to fully rule out neurological pathology.

The procedure was complicated secondary to patient's history of lumbar fusion surgery from L4-S1 twenty years previously. Scar tissue at the L3-L4 spinal space made the procedure difficult and failed three times. Pt was informed of the difficulty and he states “please Doc, try one more time.” At the insistence of the patient, a fourth attempt at lumbar puncture was made and successfully returned CSF that revealed RBC 0, WBC 2, glucose 66, and protein 73.

Furthermore, an MRI was performed that revealed enhancement of bilateral nerve roots throughout entire thoracic and lumbar spine consistent with Acute Inflammatory Demyelinating Polyneuropathy (AIDP), a form of Guillain-Barré syndrome.

The patient was admitted to ICU, underwent 5 rounds of plasmapheresis, physical, and occupational therapy, improved, and was able to ambulate out of the hospital less than 2 weeks later. He never required intubation.

## 3. Discussion and Conclusion

Guillain-Barré syndrome (GBS) is an eponym for a group of syndromes that present as either acute inflammatory or autoimmune polyradiculopathies that are often secondary to a postinfectious syndrome [1]. Variants of GBS include Miller-Fisher syndrome, acute motor (and sensory) axonal neuropathy (AMAN or AMSAN), and Acute Inflammatory Demyelinating Polyneuropathy (AIDP), along with other much rarer phenotypes. While each syndrome has unique features in their pathophysiology and symptom presentation, there are some commonalities including ascending weakness, acroparesthesia, and hyporeflexia or areflexia [1]. Infection with* C. jejuni *is thought to play a role in many cases of GBS, including the Miller-Fisher variant. Antecedent infection was present in as many as 2/3rds of patients who developed GBS [2]. Infection with* C. jejuni *causes production of antibodies targeting the lipopolysaccharides present in the outer membrane of Gram negative bacteria. These lipopolysaccharides are thought to share a similar structure to human neural antigens resulting in cross reactivity and a subsequent inflammatory neuropathy [3–5].

AMAN is an autoimmune variant where ganglioside antigens in the axon membrane itself are targeted by macrophages resulting in a Wallerian-like degeneration of the axon. Macrophages bypass the myelin sheath and attack the axolemma where the gangliosides are present [6]. Both AMAN and AMSAN are associated with an antecedent infection of* C. jejuni* and have more severe and rapid courses than AIDP. They often present with a nadir in weakness within several days along with early respiratory failure [6]. In AMAN (and AMSAN), gangliosides GM1, GM1b, GD1a, and GalNAc-GD1a are targeted [2, 7–16] by antibody mediated phagocytosis as a result of shared epitopes with* C. jejuni*. AMSAN differs from AMAN in several respects: it presents with a longer and more severe course, both sensory and motor axons are damaged, and the GalNAc-GD1a antibody is not typically present [3]. Miller-Fisher syndrome is another variant of GBS that is an autoimmune reaction with antibodies present against gangliosides GD3, Gt1a, and GQ1B [8, 9, 12, 15, 17, 18] with GQ1B being of particular clinical diagnostic significance as 90% of patients with Miller-Fisher syndrome will have a positive serology for GQ1B antibody [3]. GQ1B are present on motor nerve terminals and in vitro studies have shown that damage to these terminal will result in nerve conduction block. Miller-Fisher syndrome typically presents with areflexia, ataxia, and ophthalmoplegia [2]. AIDP is the most common variant under the GBS spectrum in North America and Europe [19]. It occurs due to a lymphocytic infiltration of the myelin sheath and damage to Schwann cell membrane components unlike AMAN and AMSAN which have damage to the axolemma. Both glycoproteins and glycolipids within the myelin sheath are damaged in a humorally mediated immune response in AIDP [19]. This results in segmental damage of the myelin sheath and subsequent nerve conduction abnormalities [20]. As opposed to AMAN, AMSAN, and the Miller-Fisher variant, data is limited regarding which, if any gangliosides are preferentially attacked in AIDP.

Diagnosis of GBS is a combination of clinical and laboratory findings; one particular instrument being used in diagnostic evaluation of GBS is the Brighton score released by the Brighton Working Group. A score of 1 is consistent with cases that meet the highest level of criteria for GBS and a score of 3 meets the minimum criteria for the diagnosis of GBS. A score of 4 or 5 indicates a case with a working diagnosis of GBS with no other likely diagnoses and one which does not have sufficient evidence to meet criteria levels 1-3 [21, 22].  Table 1 is adapted from [21].

Diagnostic criteria pertaining to all disorders within the GBS spectrum include a mostly symmetric presentation of limb weakness and a nadir in weakness anywhere from 12 hours to 4 weeks in most patients. Additional features for diagnosis of GBS may include prior infectious symptoms, cerebrospinal albuminocytological dissociation seen in cerebrospinal fluid, and the presence of distal paresthesias before onset, along with distinct electrophysiological findings [7]. With AIDP, clinical features would include hyporeflexia, areflexia, or weakness in all limbs, while supportive features would include the following electrophysiological evidence. Electrophysiological evidence of AIDP includes one of the factors in either two nerves or two factors in one nerve if all others are found to be inexcitable. The list below is adapted from [3, 22]:

AIDP:

- Distal compound action muscle potential (dcMAP) that is greater than 10% of the lower limit of normal distal motor latency that is greater than 110% of the upper limit of normal or greater than 120% if the dCMAP is less than 100% lower limit of normal.
- Motor conduction velocity <90% LLN (85% if dCMAP< 50% LLN).
- Distal motor latency >110% ULN (>120% if dCMAP <100% LLN).
- Compound muscle action potential after proximal stimulation (pCMAP) /dCMAP ratio <0.5 and dCMAP >20% LLN.
- F-response latency >120% ULN.

For AMAN and AMSAN electrophysiological criteria will not include any of the features listed in AIDP except a single demyelinating feature if dcMAP is less than 10% LLN. In AMSAN, the sensory action amplitudes will be less than the lower limit of normal while AMAN will not have any deficits in the sensory action potentials [3, 22]. Miller-Fisher syndrome will typically present with reduced sensory nerve action potentials along with an absence of the H-reflex in the soleus. Patients with Miller-Fisher will typically not have motor action potential abnormalities unlike AIDP [23].

CSF findings in GBS will typically include a high protein content with a normal white cell count that is known as albuminocytologic dissociation. Nerve inflammation and exudative pressure cause accumulation within the cerebrospinal fluid leading to elevated protein levels [24]. These findings are absent in up to 50% of patients in the first week but do typically present after the second week of illness with rates as high as 90% according to some studies [22, 25, 26]. The utility of analyzing CSF in GBS in the emergency room is low due to lack of sensitivity in early presentation [27]. White blood cell concentration in CSF above 50 cells/uL does raises suspicion for an alternative diagnosis.

Diagnostic imaging, specifically MRI, does not play a key role in the diagnosis of GBS as GBS is mainly diagnosed via clinical features and supportive electrophysiological and CSF studies; however, MRI can be used as a supplemental diagnostic modality when other supportive studies are ambiguous [28]. Findings of gadolinium enhancement of the cauda equina nerve roots on MRI evaluation have shown a sensitivity ranging from 83 to 92% in several studies in the diagnosis of GBS [29, 30]. It was demonstrated that timing did not have an effect on the results [29]. The significance of this finding may be limited due to small sample sizes of both studies. Several studies demonstrated that performing MRI on patients with GBS may be helpful when monitoring response to therapy [31, 32]. Future studies measuring MRI sensitivity in patients with early presentation (less than 1 week of symptom onset) may prove useful in evaluation, treatment, and prognosis of GBS.

Due to the declining cases of polio, GBS has now become the most common cause of acute flaccid paralysis with an annual incidence of approximately 100000 cases [1, 19]. While GBS typically progresses slowly over 2 to 4 weeks, in some instances it may have rapid onset with respiratory failure occurring within one day.

Diagnosis of GBS is often difficult as it is a rare condition with an unusual presentation and, as the aforementioned case illustrates, it is often easy to dismiss the initial presenting complaints as psychosomatic in nature [3]. Interestingly, there are often psychotic symptoms in patients with GBS [4]. In a recent prospective controlled study patients with GBS were found to have mental status changes in 31% of cases, including vivid dreams, illusions, hallucinations, and paranoid delusions [4]. Furthermore, there was statistically significant difference indicating that the psychotic symptoms exhibited by GBS patients were more severe than in cases of simple altered mental status or ICU delirium [4]. A separate prospective controlled study had corroborating findings [33], and further found that CSF albuminocytologic dissociation correlated with the occurrence of psychotic symptoms [33]. There are many case reports of patients found to have GBS who first presented with psychotic symptoms originally mistaken for psychosomatic disorders, and this mistake often leads to a delay in care [33–36].

It is imperative to remember as emergency medicine physicians that psychosomatic disorders are diagnosis of exclusion and presenting complaints must be fully evaluated. The diagnosis of GBS, particularly in the emergency room, may be missed partly due to inexperience with GBS cases, lack of spinal tap for CSF analysis, and lack of neurology consultation early in the disease process [27]. However, it is the opinion of these authors that GBS be at the forefront of an emergency medicine physician's differential diagnosis of acute weakness due to the nature of severity [20] of disease course and concern for possible respiratory failure.

## Figures and Tables

**Table 1:** Brighton diagnostic case classifications.

Level of Diagnostic Criteria	Level 1	Level 2	Level 3
Diagnostic Criteria	(i) Decreased or absent DTR's in affected limbs (ii) Time elapsed from onset to nadir from 12H to 28 days with a monophasic course (iii) Symmetric and flaccid weakness in affected limbs (iv) CSF Cell Count less than 50 per uL (v) Elevated CSF protein concentration (vi) Nerve study findings consistent with GBS variants (vii) Alternative diagnosis likely or present	(i) Decreased or absent DTR's in affected limbs (ii) Time elapsed from onset to nadir from 12H to 28 days with a monophasic course (iii) Symmetric and flaccid weakness in affected limbs (iv) CSF Cell Count less than 50 per uL^*∗*^ (v) Elevated CSF protein concentration^*∗*^ (vi) Nerve study findings consistent with GBS variants^*∗*^ (vii) Alternative diagnosis likely or present	(i) Decreased or absent DTR's in affected limbs (ii) Time elapsed from onset to nadir from 12H to 28 days with a monophasic course (iii) Symmetric and flaccid weakness in affected limbs (iv) Alternative diagnosis likely or present

^*∗*^The criterion may or may not be present.
